# Frailty classification challenges in the emergency department: agreement and variability in clinical frailty scale scoring

**DOI:** 10.1007/s40520-026-03340-4

**Published:** 2026-02-17

**Authors:** Cecilie K. Netland, Dagfinn L. Markussen, Synne Jenum, Christian Ritz, Marit S. Bakken, Harleen M.S. Grewal

**Affiliations:** 1 Department of Emergency Medicine, Haukeland University Hospital, Bergen, Norway; 2 Department of Clinical Science, Bergen Integrated Diagnostic Stewardship Cluster, Faculty of Medicine, University of Bergen, Bergen, Norway; 3 Department of Infectious diseases, Oslo University Hospital, Oslo, Norway; 4 National Institute of Public Health, University of Southern Denmark, Copenhagen, Denmark; 5 Health and Social Sciences, National Centre for Emergency Primary Health Care, NORCE Norwegian Research Centre, Bergen, Norway; 6 Department of Clinical Science, Faculty of Medicine, University of Bergen, Bergen, Norway; 7 Department of Medicine, Haraldsplass Deaconess Hospital, Bergen, Norway; 8 Department of Microbiology, Haukeland University Hospital, Bergen, Norway

**Keywords:** Frailty, Clinical frailty scale, Emergency department, Inter-rater reliability, Older adults, Pneumonia

## Abstract

**Background:**

Frailty assessment in the emergency department (ED) is essential but challenging. The Clinical Frailty Scale (CFS) is widely used, although inter-rater variability has been reported across assessors and assessment methods.

**Aims:**

To assess agreement between ED-assigned and retrospectively assigned CFS scores, and to explore characteristics associated with changes in frailty classification.

**Methods:**

We included 500 patients aged ≥ 65 years admitted with suspected pneumonia to Haukeland University Hospital (2019–2023). CFS was initially scored by nurses in the ED and reassessed retrospectively by a geriatric-trained physician using chart review. The retrospective assessment had access to broader and more objective information, including formal documentation from care services on daily function. CFS scores were categorized as fit (1–3), prefrail (4) and frail (5–9). Agreement was measured by Intraclass Correlation Coefficient (ICC).

**Results:**

CFS scores changed numerically in 252 (50.4%) patients and frailty category in 144 (28.8%). Agreement was moderate to good (ICC 0.73; 95% CI 0.68–0.77; *p* < 0.001), with retrospective assessment yielding higher scores. Agreement was highest in frail (89.7%) and lowest in prefrail patients (44.8%). Recategorized patients were older, more comorbid, more often community-dwelling with greater care dependency, and had higher 1-year mortality.

**Discussion:**

Real-time frailty assessments in the ED may underestimate frailty, particularly among patients who are community-dwelling, older, or have complex health conditions. Retrospective assessments, informed by objective documentation of functional needs, likely reflect baseline frailty more accurately.

**Conclusions:**

Frailty is often underestimated in the ED. Improved access to collateral information may improve assessment accuracy.

**Supplementary Information:**

The online version contains supplementary material available at 10.1007/s40520-026-03340-4.

## Introduction

Frailty is a state characterized by reduced physical reserves and diminished resilience against stressors [1, 2]. There are primarily two theories on the causes of frailty, the phenotype theory suggesting frailty is characterized by a specific set of physical symptoms, and the accumulations theory suggesting that frailty results from accumulation of health deficits over time [1, 3]. Consensus on definition or measurement method has not been reached [3, 4].

The global population of individuals aged > 65 years is expected to increase significantly over the next few decades, reaching an estimated 1.7 billion by 2054 [5]. Since age is strongly associated with frailty, this demographic shift is anticipated to result in a higher number of frail older adults presenting to the emergency department (ED). Frailty is a known predictor of increased hospitalization rates, longer hospital stays, cognitive impairment, disability, and higher mortality [6]. Although, there is no universally accepted gold standard for frailty assessment, comprehensive geriatric assessment conducted in a stable setting is often considered the most reliable method [7]. Early recognition of frailty in the ED can help inform treatment decisions, prioritize care, and support appropriate discharge planning [8]. In this context, the Clinical Frailty Scale (CFS) has emerged as a practical and validated tool for identifying frailty in the ED, with demonstrated predictive value for negative outcomes [6, 9].

Nevertheless, frailty assessment in the ED remains challenging. Because CFS scoring may vary based on assessor background and available data sources, it is important to explore how these factors contribute to variability in clinical practice. Notably, accurate information regarding functional status can be difficult to obtain from patients due to preexisting cognitive impairment or delirium secondary to acute illness such as community-acquired pneumonia (CAP). While several studies have reported moderate to good interrater reliability for the CFS in emergency settings (ICC 0.72–0.83), they also highlight substantial variability between raters and across visits, with a median difference of up to two CFS points [10–12]. However, other studies have found weaker agreement, with kappa values as low as 0.21 [13, 14].

The aim of this study is to compare CFS scores assessed prospectively by nurses in the ED with those retrospectively assigned by physicians through chart review, in older adults admitted with suspected pneumonia. Rather than aiming to demonstrate large discrepancies, our objective was to understand how frailty score differences arise in routine ED practice, and to explore patient groups most vulnerable to misclassification. If identified, these factors could help healthcare workers determine when to exercise greater caution or dedicate more time to frailty assessments during hospitalization.

## Method

### Study design and population

This cohort study utilizes data from three prospective studies conducted at Haukeland University Hospital (HUH) in Bergen, Norway: a feasibility study, a randomized controlled trial, and a subsequent cohort study, collectively referred to as the CAPNOR study base [15, 16]. Patient enrolment primarily occurred in the ED between December 2, 2019, and April 19, 2023. HUH serves as a primary and secondary care facility for 500,000 to 1,000,000 individuals. In Norway, patients admitted to hospital are typically first assessed by a physician in the primary healthcare system—either a general practitioner or an out-of-hours service doctor—before referral to the emergency department (ED). This gatekeeping system means that patients presenting to the ED have already been clinically evaluated and are normally admitted by an out-of-hospital physician or emergency medical services (EMS) [17].

The CAPNOR cohorts included patients aged 18 or older who presented with suspected community-acquired pneumonia (CAP) and met at least two predefined clinical criteria for CAP. Exclusion criteria included recent hospitalization (within 14 days), cystic fibrosis, severe bronchiectasis, terminal care status, or inability/unwillingness to provide a lower respiratory sample. Final discharge diagnoses were determined retrospectively based on predefined criteria in consensus meetings among investigating physicians. A full description of the diagnostic criteria applied for CAP is available in electronic supplementary material 1.

For this study, inclusion was limited to patients aged 65 or older, corresponding to the age-group for whom the CFS is validated.

As this study was based on all patients from three pre-existing cohorts, no sample size calculations were performed.

### Data collection

Patients were enrolled by nurses on weekdays between 08:00 a.m. and 09:00 p.m., typically in the ED shortly after admission. On occasions, patients were included in the wards up to 24 h post-admission. Symptoms, clinical findings, and baseline characteristics were recorded through a structured interview, review of electronic health records by study nurses, and clinical examination by the investigating physician. Additionally, results from blood tests, microbiological and radiological assessments, and the timing of any treatments, diagnosis, and discharge time were recorded. Data were securely stored in an electronic case report form (eCRF) provided by VieDoc™ (Viedoc Technologies, Uppsala, Sweden). In-house mortality rates were recorded during admission. Post-discharge mortality data was retrieved from the National Population Register.

### Frailty assessments and categorizations

Frailty was assessed using the 9-point CFS numerical score based on clinical judgment [12]. Two assessments were conducted: an initial frailty assessment performed in the emergency department (ED) by nurses (iCFS) and a retrospective frailty assessment performed by a physician with geriatric training through a systematic review of medical records (rCFS). Neither of the evaluators had previous experience in using CFS. However, all assessors received a brief, targeted orientation on how to apply the tool prior to study initiation and had access to the official CFS pictogram during assessments. The orientation materials were prepared in collaboration with a geriatrician. The physician assessor had geriatric training but had not previously applied the CFS in clinical evaluations.

### Initial frailty assessment in the ED by nurses

Nurses conducted the initial assessment (iCFS) in the ED upon admission, evaluating the patient’s pre-illness baseline, typically 14 days before illness onset. This assessment primarily relied on patient interview and information from next of kin, if present in the ED.

### Retrospective frailty assessment by physician

All patients subsequently underwent a retrospective frailty assessment (rCFS) performed in 2024 by a physician with geriatric training (C.K.N.) by systematically reviewing medical records from the time of admission, ensuring that the assessment reflected patient’s pre-admission frailty status rather than subsequent clinical changes. This assessment incorporated a systematic electronic chart review utilizing multiple sources, including electronic reports from municipal care facilities or home care. In Norway, healthcare services, including hospitals, nursing homes, and home nursing are publicly managed. For patients receiving home nursing, the municipal care facility provides an electronic report on the patient’s functional status, typically available within 24 h after admission. Where available, this report was essential for assessing pre-admission functional status. Additional sources were recent outpatient assessments and hospital admissions and documented information from relatives or staff during the current admission. If data were limited, and the patient was neither multimorbid nor described as fit or exercising regularly, a default score of 3 was assigned. In complex cases, evaluations were discussed with senior physicians (including a specialist in infectious diseases and emergency medicine (D.L.M.) and a specialist in internal and geriatric medicine (M.S.B.) to reach a consensus. If uncertainty remained, the higher score was assigned (e.g., if unsure whether the correct score was 4 or 5, a score of 5 was recorded). The evaluating physicians were not blinded to post-admission outcomes (e.g. readmissions, deaths).

For the rCFS assessments, patients with underlying schizophrenia (*n* = 2) and intellectual disabilities (*n* = 3) were assessed using the same classification principles as those with dementia (e.g. degree of frailty corresponds with degree of disease/disability), following the guidance in the official CFS manual [18].

In our design, the rCFS was considered the reference (gold-standard) assessment, owing to its access to richer and more comprehensive data sources. Agreement between iCFS and rCFS was therefore evaluated relative to this reference, allowing identification of potential over- or underestimation in the initial ED assessment.

For statistical analysis, CFS scores were categorized into three frailty groups: fit (CFS 1–3), prefrail (CFS 4), and frail (CFS 5–9). Additionally, patients were categorized based on a change in CFS classification (e.g. fit, prefrail, frail) from iCFS to rCFS. This categorization - the “changed category” group vs. the “unchanged category” group - was used in analyses aiming to identify characteristics that may complicate frailty assessment or increase the probability of an incorrect score. The “changed category» included patients whose frailty status shifted between categories — for example, a patient moving from CFS 3 (fit) to CFS 4 (prefrail) would be in the “changed category”. In contrast, the “unchanged category” consisted of patients whose (numerical) CFS scores may have fluctuated, but their frailty status remained within the same category. For instance, a patient moving from CFS 5 to 6 (both classified as frail) would be in the “unchanged category” for this analysis.

### Statistical analysis

Patient characteristics were summarized using means ± SD or medians [25th to 75th percentiles] for continuous variables and frequencies (percentage) for categorical variables. The Charlson Comorbidity Index (CCI) was employed to quantify comorbid conditions. The CFS, which ranges from 1 to 9, was analysed both as a continuous variable and as a categorical variable by grouping patients into three categories: fit (CFS 1–3), pre-frail (CFS 4), and frail (CFS 5–9). Agreement between the initial (iCFS) and retrospective (rCFS) frailty assessments was evaluated using the Intraclass Correlation Coefficient (ICC) for single measurements. A correlation coefficient greater than 0.9 is considered excellent, values between 0.9 and 0.75 are good, between 0.75 − 0.5 are moderate and less than 0.5 are poor [19]. The mean differences in CFS scores were visualized with a Bland–Altman plot, while transitions between frailty groups were depicted using a Sankey diagram. A p-value of < 0.05 was considered statistically significant.

The categorical variables were compared using the chi-square or Fisher’s exact test where appropriate, while continuous variables were assessed using either the t-test or the Mann-Whitney U-test, depending on their distribution. Patients who demonstrated a change in frailty group classification (e.g., from fit to pre-frail) were compared to those whose classification remained unchanged.

IBM SPSS Statistics version 29.0 and R version 4.4.1 were used for all statistical analyses [20, 21].

## Results

**Fig. 1:**
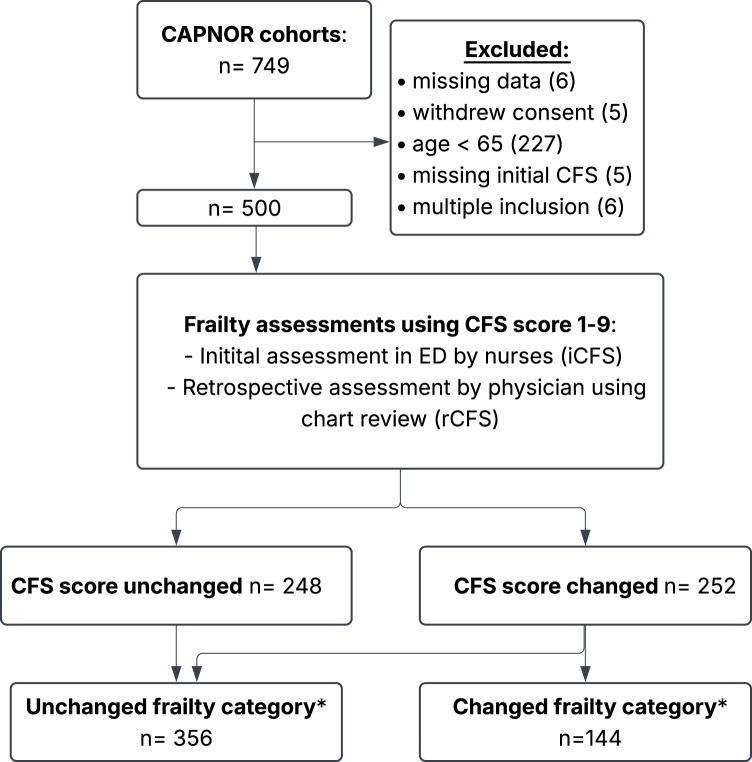
Flow chart illustrating participant inclusion, frailty assessment process and categorization in a Norwegian ED

Of the 749 patients enrolled in the CAPNOR study cohort, 500 were eligible for this study and underwent a second, retrospective assessment using the CFS (rCFS). Patient selection and exclusions are illustrated in Fig. 1. Average age in years was 77.5 years, and 221 (44.2%) were women. Recent weight loss occurred in 139 (27.8%) of the patients, 79 (15.9%) had been admitted to a nursing home in the last twelve months, and 17 (3.4%) were admitted directly from a nursing home. Preexisting cognitive impairment was found in 23 (4.6%) of the patients. The median National Early Warning Score (NEWS) was 5 (IQR 3–7). Detailed baseline characteristics of the 500 patients are provided in Table 1. In 85 patients, data on height and/or weight were missing, which prevented the calculation of body mass index (BMI). Data for 68 of these 85 patients were retrieved through chart reviews within a ± two-year window from inclusion, leaving 17 patients with missing BMI data. Additionally, one patient was missing a NEWS score, which was retrospectively manually recorded from inclusion time. Missing values for recent weight loss and prior nursing home admissions are also noted, listed in Table 1.

Figure 1 Flow chart illustrating participant inclusion in a Norwegian ED, frailty assessment and categorization. The CAPNOR cohorts include patients from three prospectives studies at Haukeland University Hospital (2019–2023), comprising adult (> 18 years) admitted with suspected pneumonia. Abbreviations; *ED*: Emergency Department; *CFS*: Clinical Frailty Scale. *Frailty categories: fit (CFS 1–3), prefrail (CFS 4) and frail (CFS 5–9).

**Table 1:** Baseline characteristics for Norwegian ED patients with suspected pneumonia

Demographics	*n* = 500
Age (mean ± SD)	77.5 ± 7.8
Sex, female	221 (44.2%)
BMI^*^ (median [Q1-Q3])	24.2 [21.2-27.4]
Recent weight loss †	139 (27.8%)
Nursing home admission^‡^	
> 12 months earlier	58 (11.7%)
2-12 months earlier	42 (8.5%)
Within last month	37 (7.5%)
Smoking, pack years (median [Q1-Q3])	27 [0.0-50.0]
**Level of care**	
*At admission*	
No formal care	356 (71.2%)
Receiving health care services	127 (25.4%)
Institution	17 (3.4%)
*At discharge*	
No formal care	294 (58.8%)
Receiving health care services	107 (21.4%)
Institution	83 (16.6%)
Died during admission	16 (3.2%)
**Severity scales**	
NEWS score at admission (median [Q1-Q3])	5 [3-7]
CRB65 score at admission (median [Q1-Q3])	1 [1-2]
**Frailty assessments**	
iCFS score (median [Q1-Q3])	3 [2.5-4]
rCFS score (median [Q1-Q3])	4 [3-5]
Changed numerical CFS score	252 (50.4%)
**Discharge diagnosis**	
Pneumonia	337 (67.4%)
Other respiratory infection	87 (17.4%)
COPD-exacerbation (non-infectious)	19 (3.8%)
Other	57 (11.4%)
**Comorbidity**	
Charlson Comorbidity Index (CCI) (median [Q1-Q3])	4.0 [3-6]
Any chronic disease	476 (95.2%)
COPD	229 (45.8%)
Coronary disease	128 (25.6%)
Hypertension	240 (48.0%)
Atrial fibrillation/flutter	115 (23.0%)
Stroke/TIA	64 (12.8%)
Chronic kidney disease	67 (13.4%)
Heart failure	103 (20.6%)
Diabetes	73 (14.6%)
Cancer	57 (11.4%)
Psychiatric disease	75 (15.0%)
Cognitive impairment	23 (4.6%)
**Length of stay (days)** (median [Q1-Q3])	3.9 [2.1-6.1]
**Mortality**	
1 year	98 (19.6%)

^*^ Missing values: 17, *n* = 483

^†^ Missing values: 84, *n* = 416

^‡^ Missing values: 4, *n* = 496

Recent nursing home admission: admission within the last twelve months; recent weight loss: > 5% weight loss within the previous 6 months; institution: nursing home, hospital, palliative care centre or short-term health care facility like Covid-clinics

BMI; Body Mass Index; NEWS: National Early Warning Score; *CRB65*: Confusion, Respiration rate, Blood pressure, Age > 65; *CFS*: Clinical Frailty Scale; COPD: Chronic Obstructive Pulmonary Disease; TIA: Transient ischemic attack

### Concordance between initial and retrospective CFS assessments

**Fig. 2:**
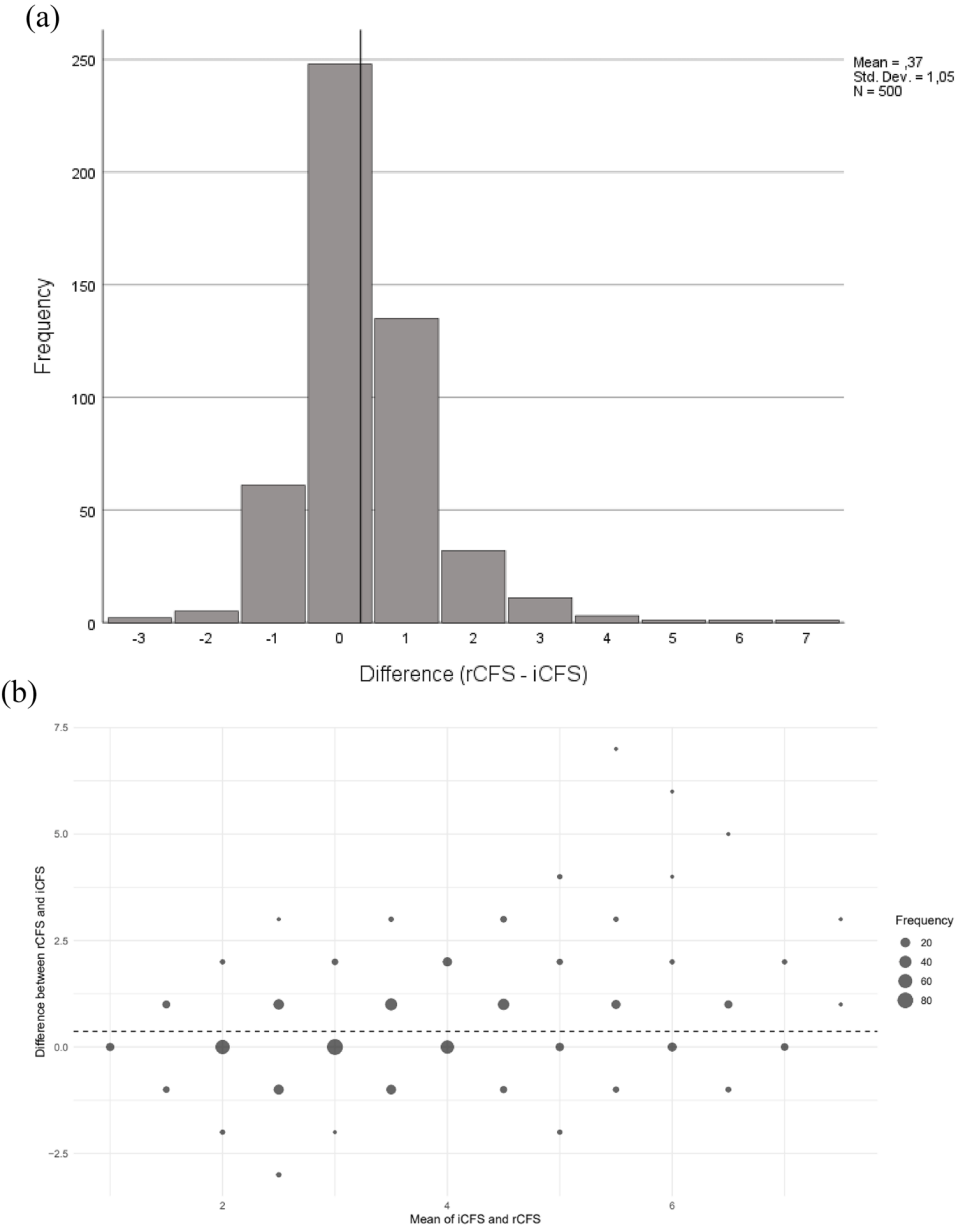
Distribution of Differences Between Initial and Retrospective CFS Scores (2a) and Bland-Altman Plot of Agreement Between Initial and Retrospective CFS Assessments (2b). (**a**) Histogram displaying the frequency of difference in scores between the initial Clinical Frailty Scale score (iCFS) assessed by nurses in a Norwegian emergency department and retrospective score (rCFS) assigned by a physician through chart review. Abbreviations; CFS: Clinical Frailty Scale; ED: Emergency Department. (**b**) Bland-Altman plot illustrating agreement between the initial Clinical Frailty Scale (iCFS) and retrospective Clinical Frailty Scale (rCFS) assessments. The dotted line represents the mean difference. As the mean frailty score increases, the variability in differences between assessments also increases, indicating greater disagreement at higher frailty levels. Abbreviations; CFS: Clinical Frailty Scale; ED: Emergency Department

Among the 500 patients, 252 (50.4%) had a changed numerical CFS score in the second, retrospective frailty assessment, rCFS. A discrepancy of more than one point in the rCFS score was observed in 56 (11.2%). The average change was 0.37 points (95% CI: 0.27 to 0.45), as shown in Fig. 2a, which illustrates the frequency distribution of frailty score differences between the initial CFS score (iCFS) and rCFS score. The agreement between iCFS and rCFS frailty assessments, measured by the intra-class correlation coefficient (ICC), was 0.73 (95% CI: 0.69–0.77; *p* < 0.001), indicating moderate to good agreement. Figure 2b shows the mean difference between the two frailty assessments and the frequency of each difference, highlighting the overall agreement, with the largest differences observed in the higher frailty scores.

**Fig. 3:**
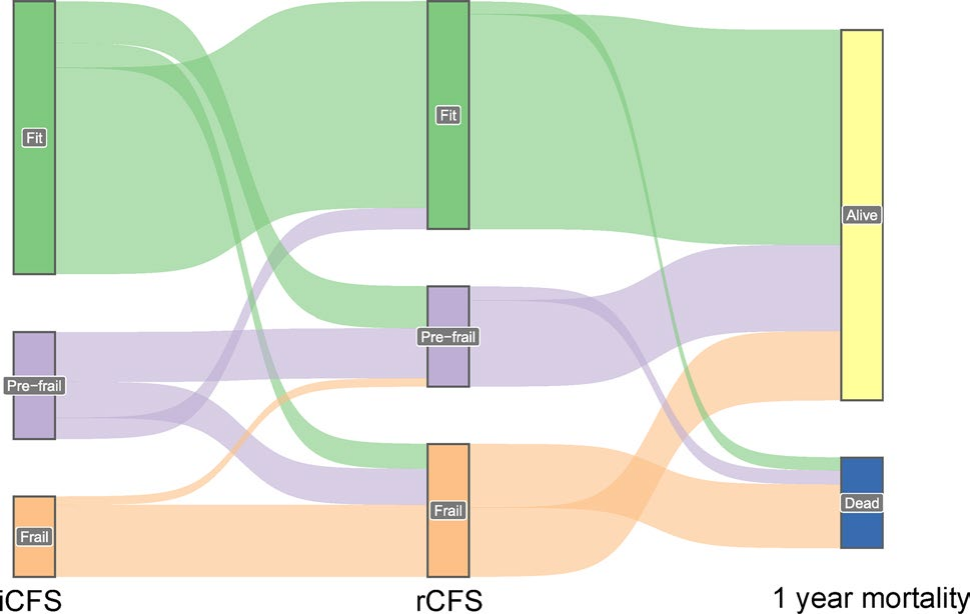
- Illustration of agreement between initial and retrospective CFS scores in ED patients with suspected pneumonia

Among patients initially classified as fit (CFS 1–3), the agreement between initial and retrospective assessment was good, with 75.4% remaining “fit” after the retrospective assessment. In the prefrail (CFS 4) category, the agreement was 44.8%, with notable reclassification into “fit” (19.9%) and “frail” (CFS 5–9) (35.3%). The highest agreement was achieved for patients classified as “frail,” at 89.7%. Agreement in the different frailty assessments by *frailty categories* is represented as numbers in Table 2 and graphically in Fig. 3.

**Table 2:** Agreement statistics between initial and retrospective CFS frailty categories in ED patients with suspected pneumonia

Retrospective CFS category (rCFS) iCFS category	Fit	Prefrail	Frail	Agreement Rate (%)
Fit (CFS 1–3)	224	45	28	75.4%
Prefrail (CFS 4)	23	52	41	44.8%
Frail (CFS 5–9)	0	9	78	89.7%
**Total**	247	108	144	

Agreement between initial (ED-based) (iCFS) and retrospective (chart review-based) (rCFS) frailty assessments varied across frailty categories. Abbreviations; *CFS*: Clinical Frailty Scale; *ED*: Emergency Department; *iCFS*: Initial Clinical Frailty Scale score; *rCFS*: retrospective Clinical Frailty Scale score

Figure 3. Sankey plot illustrating patient transitions between frailty categories - fit, prefrail and frail - from the initial Clinical Frailty Scale (iCFS) assessments by nurses in the ED, to retrospective CFS (rCFS) assessments by physicians via chart review, and one-year mortality outcomes. The diagram displays how frailty status at each assessment relates to survival. Frailty categories: fit (CFS 1–3), prefrail (CFS 4) and frail (CFS 5–9). Abbreviations; *CFS*: Clinical Frailty Scale; *ED*: Emergency Department. *iCFS*: Initial Clinical Frailty Scale score; *rCFS*: retrospective Clinical Frailty Scale score.

### Patient characteristics associated with change in CFS category

Several statistically significant differences were observed between patients whose frailty category changed versus those with unchanged categories (Table 3). The changed group had a higher mean age (79.2 vs. 76.8 years, *p* = 0.002), higher NEWS score (median 6 vs. 5, *p* = 0.024), and higher levels of comorbidity measured by the Charlson Comorbidity Index (CCI) (median 5 vs. 4, *p* < 0.001). Additionally, the changed group showed higher prevalence of chronic obstructive pulmonary disease (COPD) (59.7% vs. 40.2%, *p* < 0.001), chronic kidney disease (18.8% vs. 11.2%, *p* = 0.026), psychiatric disorders (20.1% vs. 12.9%, *p* = 0.041), and cognitive impairment (7.6% vs. 3.4%, *p* = 0.039), underweight status (13.2% vs. 6.2%, *p* = 0.010), recent weight loss (35.4% vs. 24.7%, *p* = 0.049), and greater receipt of healthcare services (40.3% vs. 19.4%, *p* < 0.001). One-year mortality was also higher (25.7% vs. 17.1%, *p* = 0.029). Conversely, the unchanged group had a higher proportion of overweight patients (50.8% vs. 39.6%, *p* = 0.022) and more patients living without formal care (76.7% vs. 57.6%, *p* < 0.001).

**Table 3:** Baseline characteristics by change in frailty category (CFS) in Norwegian ED pneumonia patients, *n*= 500

Demographics	Unchanged frailty category (*n*=356)	Changed frailty category (*n*=144)	*p*-value
Age (mean ± SD)	76.8 ± 7.8	79.2 ± 7.5	0.002
Sex, female	152 (42.7%)	68 (47.2%)	0.356
BMI^*^ (median [Q1-Q3])	24.4 [22.0-27.5]	23.0 [19.4-27.1]	0.002
Overweight	181 (50.8%)	57 (39.6%)	0.022
Normal weight	153 (43.0%)	68 (47.2%)	0.387
Underweight	22 (6.2%)	19 (13.2%)	0.010
Recent weight loss^†^	88 (24.7%)	51 (35.4%)	0.049
Recent nursing home admission^‡^	50 (14%)	29 (20.1%)	0.091
Smoking, pack years (median [Q1-Q3])	24.4 [0.0-45.0]	35 [4.5-50.0]	0.095
**Level of care**			
*At admission*			
No formal care	273 (76.7%)	83 (57.6%)	<0.001
Receiving health care services	69 (19.4%)	58 (40.3%)	<0.001
Institution	14 (3.9%)	3 (2.1%)	0.302
*At discharge*			
No formal care	234 (65.7%)	60 (41.7%)	<0.001
Receiving health care services	59 (16.6%)	48 (33.3%)	<0.001
Institution	53 (14.9%)	30 (20.8%)	0.106
Died during admission	10 (2.8%)	6 (4.2%)	0.435
**Severity scales**			
NEWS score (median [Q1-Q3])	5 [3-6]	6 [3-7]	0.024
CRB65 score (median [Q1-Q3])	1 [1-2]	1 [1-2]	0.246
**Frailty assessments**			
iCFS score (median [Q1-Q3])	3 [2-4]	3 [3-4]	0.040
rCFS score (median [Q1-Q3])	3 [2-4]	4 [4-5]	<0.001
Changed numerical CFS score	108 (42.9%)	144 (57.1%)	<0.001
**Discharge diagnosis**			
Pneumonia	245 (68.8%)	92 (63.9%)	0.287
Other respiratory infection	60 (16.9%)	27 (18.8%)	0.613
COPD-exacerbation (non-infectious)	11 (3.1%)	8 (5.6%)	0.192
Other	40 (11.2%)	17 (11.8%)	0.856
**Comorbidity**			
Charlson Comorbidity Index (CCI) (median [Q1-Q3])	4.0 [3-5]	5.0 [4-6]	<0.001
Any chronic disease	333 (93.5%)	143 (99.3%)	0.006
COPD	143 (40.2%)	86 (59.7%)	< 0.001
Coronary disease	85 (23.9%)	43 (29.9%)	0.165
Hypertension	167 (46.9%)	73 (50.7%)	0.443
Atrial fibrillation/flutter	75 (21.1%)	40 (27.8%)	0.106
Stroke/TIA	43 (12.1%)	21 (14.6%)	0.448
Chronic kidney disease	40 (11.2%)	27 (18.8%)	0.026
Heart failure	70 (19.7%)	33 (22.9%)	0.415
Diabetes	53 (14.9%)	20 (13.9%)	0.775
Cancer	39 (11.0%)	18 (12.2%)	0.623
Psychiatric disease	46 (12.9%)	29 (20.1%)	0.041
Cognitive impairment	12 (3.4%)	11 (7.6%)	0.039
**Length of stay (days)** (median [Q1-Q3])	3.6 [2.1-6]	4.0 [2.5-7]	0.123
**Mortality**			
1 year	61 (17.1%)	37 (25.7%)	0.029

^*^ Missing values: 17, *n* = 483

^†^ Missing values: 84, *n* = 416

^‡^ Missing values: 4, *n* = 496

Recent nursing home admission: admission within the last twelve months; recent weight loss: > 5% weight loss within the previous 6 months; institution: nursing home, hospital, palliative care centre or short-term health care facility like Covid-clinics

ED: Emergency Department; CFS: Clinical Frailty Scale; BMI; Body Mass Index; NEWS: National Early Warning Score; CRB65: Confusion, Respiration rate, Blood pressure, Age > 65; iCFS: Initial Clinical Frailty Scale; rCFS: Retrospective Clinical Frailty Scale; COPD: Chronic Obstructive Pulmonary Disease; TIA: Transient ischemic attack

## Discussion

This study demonstrates that Clinical Frailty Scale (CFS) scores assigned by nurses in the ED differs significantly from those performed by physicians with geriatric training through chart reviews. Despite this, we observed moderate to good overall agreement between the two assessments (ICC 0.73). Retrospective assessments were more likely to assign higher CFS scores. Agreement was highest for frail patients (89.7%) and lower for prefrail patients (44.8%). CFS scores changed numerically in 50.4% of patients, and 28.8% were reclassified into a different frailty category. In this study, we used the term “changed numerical CFS score” to describe any numerical difference between iCFS and rCFS assessments (*n* = 252), while “changed frailty category” refers to patients who crossed from one frailty group (fit, prefrail, frail) to another (*n* = 144). These changes were associated with several patient characteristics, most notably level of care, comorbidity burden, and the presence of COPD.

Retrospective assessments, benefited from structured reports describing patients’ baseline function and care needs, particularly for those receiving municipal home nursing. These reports were often available within the first 24 h of admission, but not accessible at the time of the ED evaluation. Retrospective assessments also incorporated input from next of kin and ward staff obtained later during hospitalization. These additional sources likely contributed to higher frailty scores and were considered more accurate reflections of patients’ baseline function. While some discordance between the two assessment types was expected due to differences in timing and information availability, our findings illustrate the real-world challenges of performing reliable frailty assessments in acute care.

Our study suggests that frailty assessments conducted in the ED may systematically underestimate frailty. This likely reflects the limitations of the ED setting, where assessments often rely on brief clinical encounters, limited collateral information, and patient or caregiver reports. Under-reporting—due to acute illness, cognitive impairment, or lack of insight—may further reduce accuracy. Patients categorized as frail in the ED, were rarely reclassified, suggesting that ED-based assessments may be sufficient to identify overt frailty, but are less reliable in borderline cases. The results demonstrated that the agreement between the assigned categories was lowest for patients assigned to the pre-frail category in the ED. Several factors may explain this finding. First, CFS 4 represents only a single numerical level, whereas the “fit” and “frail” category each include several (CFS 1–3 and CFS 5–9, respectively). This narrower range inherently increases the probability of reclassification. In addition, the descriptive criteria for CFS 4 likely covers a heterogeneous group of individuals who function well but display early signs of vulnerability, which might be easily missed in a busy ED. Borderline cases like these often involve patients who alternate between independence and dependency, or whose function decline is subtle and situational. In clinical practice—particularly in the fast-paced ED environment—CFS 4 may be assigned when the assessor senses that a patient is no longer fully fit but lacks sufficient information to determine whether frailty, or in which degree, is already established. Consequently, the prefrail category may act as both a diagnostic “grey zone” and a practical default, contributing to the observed variability between initial and retrospective assessments.

Rather than invalidating ED-based assessments, these differences highlight modifiable factors—such as improved access to information—that may improve consistency and clinical utility. While the CFS is considered a practical tool for frailty assessment in acute care, its reliability is influenced by the assessor’s familiarity with the scale and access to adequate collateral information. To improve consistency, structured training modules and educational resources have been developed to improve inter-rater agreement and promote more consistent use of the CFS in clinical practice [22, 23]. Improving the accuracy of frailty classification is not only a methodological goal, but also a clinical priority, particularly given the growing emphasis on frailty-informed care.

Frailty assessments are increasingly recommended in clinical care. In Norway, recent publications have proposed implementing routine frailty assessments in emergency departments, and the European Joint Action on Frailty (ADVANTAGE) recommends evaluating frailty during all healthcare encounters involving older patients [24, 25]. Identifying pre-frail patients is crucial, as early interventions can prevent progression to frailty and reduce the risk of adverse outcomes during hospitalization. In this context, accurate classification of frailty serves primarily to inform clinical decision-making and risk stratification at the point of care.

Several studies have validated CFS assessment conducted via chart review [26–28]. In our study, retrospective assessments from chart review yielded significantly higher CFS scores. This contrasts with a study conducted in Sweden that reported significantly lower retrospective scores when assessed by a medical student, while a study from Germany reported only a slight underestimation of frailty in retrospectively assigned CFS scores [26, 28]. These differences likely reflect variability in assessor training, data completeness, and interpretation of scoring thresholds across studies. In our study, the retrospective assessors had access to structured care reports from the national health system, including municipal home nursing documentation, which is routinely shared across care levels in Norway. This integrated data infrastructure likely contributed to more comprehensive assessments and may not be directly comparable to settings with less centralized or fragmented healthcare systems.

To our knowledge, few studies have examined which patients are particularly challenging to assess with the CFS. In the previously mentioned Swedish study, the authors compared patients with concordant CFS scores (no change between assessors) to those with discordant scores (any numerical change between assessments) [26]. Their sample was smaller (*n* = 145), and they defined discordance as any numerical difference in score. In contrast, we focused on shifts between frailty categories, which are more likely to impact clinical decision-making. We found that increasing age and greater comorbidity were associated with changes in frailty classification. Community-dwelling patients receiving healthcare services also posed challenges, likely due to their clinical heterogeneity and limited availability of collateral information in the ED. These patients may range from fully independent to highly dependent on family support, complicating accurate baseline assessment due to a lack of detailed knowledge about the extent of support required in daily life. In contrast, agreement was higher for patients admitted from or discharged to institutions where functional status is more consistently documented. Disease severity also appeared to influence frailty scoring. COPD, chronic kidney disease and higher NEWS score were significantly associated with frailty reclassification, though underlying mechanisms remain uncertain. A higher proportion of underweight patients in the changed category group, and overweight patients in the unchanged group, may further reflect differences in underlying frailty. Lastly, we observed a higher one-year mortality in patients whose frailty category changed between assessments. This relationship will be explored in greater detail in a forthcoming analysis looking in on the impact of frailty on mortality in CAP patients.

A major strength of this study is its relatively large sample size, use of prospectively enrolled cohorts and a real-world ED setting. By comparing frailty assessments from multiple healthcare professionals using different approaches, the study provides a realistic representation of clinical practise and enhances the generalizability of the findings to routine ED care. The inclusion of structured documentation from municipal care services in retrospective assessments was particularly valuable for capturing objective information on baseline function, especially in patients receiving home nursing.

Although several factors, such as comorbidity, care level, and specific conditions like COPD, were associated with changes in frailty classification, the study was not designed to identify independent predictors. Key variables, including acute delirium and weight loss, were inconsistently documented, limiting the feasibility of meaningful multivariable analysis. For this reason, we chose not to perform such analyses. Instead, our findings support the hypothesis that certain patient characteristics and acute clinical factors may contribute to misclassification of frailty status during hospitalization. We therefore consider these results hypothesis-generating and recommend that future studies be designed specifically to test this hypothesis and improve assessment protocols in acute care.

Several limitations should be acknowledged. First, there is a risk of information bias. The physician performing the retrospective CFS (rCFS) assessments was not blinded to patient outcomes, including readmissions and mortality —which may have introduced bias —potentially leading to higher frailty scores assigned in cases with poor outcomes. Although the rater was instructed to base each score strictly on pre-admission functional information, this bias cannot be entirely excluded and should be considered when interpreting the results. Second, inter-rater and methodological limitations should be considered. Differences in timing, assessor training, and methodology between the initial and retrospective assessments complicate direct comparisons. Although both the nurses and the physician assessor received instructions on how to apply the CFS, neither had previous experience with the tool and this may have introduced variability. The physician gained substantial experience over the course of the retrospective assessment, potentially reducing intra-rater variability and improved scoring consistency over time. Nurses preformed frailty scoring alongside multiple other tasks and frailty assessment was not their primary focus, which may have led to inaccurate frailty scoring. Some rCFS assessments, particularly for patients with severe COPD or psychiatric illness, required additional input from senior clinicians, which may not reflect routine practice. These factors together reflect inter-rater variability inherent to real-world use of the CFS. Third, sampling limitations may affect generalizability. Our study included only patients admitted with suspected pneumonia and may not be generalizable to other populations. Most patients were initially assessed by out-of-hospital physicians or EMS, which limits applicability to settings where patients present directly to the ED. Patients admitted at night were underrepresented, and informed consent requirements likely excluded those with moderate-to-severe dementia or delirium. Additionally, parts of the study were conducted during the early phase of the COVID-19 pandemic, which may have further restricted access to collateral information and affected frailty classification

In summary, our findings highlight real-word challenges of frailty assessment in acute care, particulary limited access to collateral information, which may result in underestimation of frailty in the ED.

## Conclusion

The Clinical Frailty Scale is a practical tool for emergency settings, but frailty may be underestimated in the ED, most importantly due to limited access to collateral information. Our findings point to modifiable factors to improve assessment consistency, increased awareness of rater bias, and systematic access to care documentation. Targeted baseline assessments should be given to patients with CFS 3–5, those receiving community healthcare, and individuals with complex comorbidities especially COPD, who may be at higher risk of misclassification.

## Supplementary Information

Below is the link to the electronic supplementary material.

Supplementary Material 1

## Data Availability

The raw datasets generated and analysed during the current study are not publicly available due to privacy concerns under the European General Data Protection Regulation (GDPR). However, de-identifed participant data and aggregated data presented in this study can be made available from the corresponding author upon reasonable request. Data sharing will be contingent on the receiving institution’s agreement to a Data Use Agreement.
