# Supplementary material for: Frailty classification challenges in the emergency department: agreement and variability in clinical frailty scale scoring
Source: Aging Clin Exp Res. 2026 Feb 17;38(1):87. doi: 10.1007/s40520-026-03340-4 (PMC12960408; doi:10.1007/s40520-026-03340-4)
Supplement: Supplementary file 1 — Supplementary Material 1 [file 40520_2026_3340_MOESM1_ESM.docx]

**Diagnostic Criteria for Community-Acquired Pneumonia in the CAPNOR Cohorts**

**Overview**

This Electronic Supplementary Material (ESM) provides a detailed description of the inclusion, exclusion and diagnostic criteria used in the CAPNOR cohorts. These details complement the abbreviated methodology presented in the main article.

**Inclusion Criteria**

Patients were eligible if they met all of the following:

1. **Age ≥18 years**
2. **Presentation with suspected community-acquired pneumonia (CAP)**
3. **Fulfilment of at least two predefined clinical criteria**, including:
   - New or worsened cough
   - Expectoration
   - Dyspnoea
   - Haemoptysis
   - Pleuritic chest pain
   - Fever ≥38°C
4. **Radiological or auscultatory findings consistent with pneumonia**

These criteria were consistent across all CAPNOR cohorts.

**Exclusion Criteria**

Patients were excluded if any of the following applied:

- Hospital admission within the preceding 14 days
- Cystic fibrosis
- Severe bronchiectasis
- Terminal care status
- Inability or unwillingness to provide a lower respiratory sample

*Note:* In the third CAPNOR cohort, the exclusion window for recent hospitalization was adjusted to permit inclusion of patients admitted within the preceding 14 days.

**Diagnostic Definitions**

Final discharge diagnosis were determined retrospectively based on predefined criteria in consensus meetings among investigating physicians. CAP was defined as a clinical diagnosis of suspected pneumonia documented by the treating physician and supported by at least two diagnostic criteria and without evidence of an alternative non-respiratory source of infection. Patients were categorized into clinical CAP, radiologically confirmed CAP and other respiratory tract infections or diagnoses, based on a combination of patient history, imaging results, and laboratory findings. Other diagnoses were obtained from discharge summaries.

**1. Clinical CAP**

Clinical CAP required:

- In-hospital treatment or diagnosis confirmed by a study investigator
- Supported by at least two diagnostic criteria

**2. Radiologically Confirmed CAP**

Radiologically confirmed CAP required:

- Meeting criteria for clinical CAP
- Presence of a new or increased infiltrate on chest radiograph or CT scan

Diagnostic criteria included:

- New or worsened cough
- Purulent sputum or change in sputum character
- Fever or hypothermia
- Auscultatory findings consistent with pneumonia
- Leukocytosis or neutrophilia
- Elevated C-reactive protein (CRP >50 mg/L)
- New or worsening dyspnoea, tachypnoea, or hypoxemia
